# Blurring boundaries or structural coupling? Revisiting the science-medicine interface through the analogy of politics and law

**DOI:** 10.3389/fsoc.2026.1872166

**Published:** 2026-07-28

**Authors:** Dominik Hofmann

**Affiliations:** Faculty of Sociology, Bielefeld University, Bielefeld, Germany

**Keywords:** biomedicine, law, medicine, politics, science, systems theory

## Abstract

Recent developments in cutting-edge medicine have inspired numerous analyses claiming the boundaries between scientific research and medical practice are blurring. This paper offers an alternative description of the dynamics at stake. Drawing on Luhmann's systems theory, it analyzes the relationship between medicine and biomedical science as a historically evolved pattern of structural coupling between two operationally closed functional systems. Methodologically, it employs systems comparison, using the well-developed systems theoretical description of the law-politics relationship as a template for examining analogies to the relation between medicine and science. Through a reconstruction of their center/periphery-differentiation, contingency formulae, structural coupling, and conditional programs, it shows that both constellations are marked by a distinctive arrangement of mutual externalization of legitimation. A historical account of the co-evolution of medicine and law further clarifies why medicine ultimately externalized the justification of its decision programs to science. Against this background, contemporary biomedical transformations are interpreted as signs of strain on established coupling mechanisms rather than as evidence of systemic fusion. The blurring boundaries debate thus appears as a symptom of intensified interdependence under conditions of scientific innovation to which the medical system is forced to react.

## 1 Introduction

Sickle cell anemia is a genetic disorder, named after the abnormal form of red blood cells in affected individuals. It causes the blood cells to block the capillaries through which they normally flow freely. In modern medicine, the disease is notorious, not so much for pathological or epidemiological reasons as for the role the discovery of its underlying mechanism played in the development of the field (Morange, 2020, p. 124f.). In 1949, researchers first showed that the disease follows Mendelian patterns of genetic inheritance and then that its occurrence is linked to an abnormal hemoglobin structure, before protein sequencing revealed in 1956 that this abnormality results from the substitution of a single amino acid in a peptide. For the first time, a small genetic change could be considered to explain a pathology. The process leading from a gene mutation to disease onset is complicated, but in the case of sickle cell anemia it is not complex. It can be traced through relatively well-defined causal chain^1^. By contrast, other diseases with a strong molecular basis, such as cancer or auto-immune diseases, are indeed considered “complex diseases”, since their underlying molecular alterations are multiple, interact non-linearly, spread over different levels of organismic organization, and experience feedback effects (Mossman, 2014). Sickle cell anemia's “simple” pathogenesis allows relatively straightforward diagnosis with a genetic test. Therapy, however, is a different issue, especially curative treatment. While bone marrow transplantation has been an option since 1984, it took many further discoveries, among them in stem cell therapy and genetic editing, as well as a long drug development and approval process, before gene therapy targeting the molecular pathway of hemoglobin production could for the first time be used, in a children's hospital in 2024^2^ To enable similar progress with “complex diseases,” millions of patients worldwide are inscribed in clinical studies that seek correlations between candidate drug components and therapeutic effects. As sociologists of science and medicine have long pointed out, it is not easy, in modern biomedicine, to clearly distinguish scientific research from medical care practices (Cambrosio et al., 2018; Latimer et al., 2006; Timmermans, 2010). Recently, the diagnosis of “blurring boundaries” between the two fields has gained even more prominence, with the development of subfields such as “precision” and “systems medicine,” where interdisciplinary teams of experts from nursing, pathology, microbiology, genetics etc. collaborate in specialized health centers in which most or even all patients are also registered as trial participants (Kerr et al., 2021; Nelson et al., 2014; Wainwright et al., 2006; Hallowell et al., 2009; Lewis et al., 2014; Petty and Heimer, 2011). The blurring boundaries diagnosis has turned into a staple in, mainly ethnography-based, analyses of innovative biomedical research.

Given this situation, systems theory appears, at first glance, to offer a strong counterpoint. It not only defines medicine and science as two autonomous, operationally closed functional systems, but also provides clear criteria for describing the boundaries that other theoretical approaches perceive as blurred. In systems theory^3^, functional systems consist of communications linked together by their shared reference to a guiding distinction (true/untrue for science, ill/health for medicine), with decision rules (“programs”) determining which of the two values applies in a given situation. As a consequence of this arrangement, neither professional nor client roles (overlapping in the hybrids “clinician-scientist” and patient-trial participant), nor the physical spaces of laboratories and hospitals (now converging in computer systems and clouds that record health data), nor the intervention into a patient's body (which can be simultaneously serve therapeutic purposes and research data-generation) define the systemic boundaries (or their dissolution). Rather, medicine and science are distinguished by their respective logics: finding true and exposing false statements and turning ill patients back into healthy individuals, each according to system-specific rules and procedures. From this perspective, biomedicine has not broken down any boundaries. Employing the concept of operational closure through coded and programmed communication, systems theory offers a framework that clarifies the nature of the boundaries, within which concepts such as hybrid roles, boundary work, and boundary permeability—used to describe the empirical reality of current biomedical research—can be analytically situated.

On closer inspection, however, systems theory's own demarcation of the functional system of medicine is less certain than its confident abstraction of structural principles might suggest. This starts with Luhmann's own shifting terminology, which alternates between “medicine,” “system of medical care,” “system of patient care”, “health system”. It extends further into the (relatively limited) body of systems-theoretical literature that followed in the wake of Luhmann's writings on medicine. Most of this post-Luhmannian work on medicine has focused on what the system comprises and what it excludes. Several authors argue that the focus on care for ill patients is too narrow (Bauch, 1996; Hafen, 2009, 2015). Newer developments in the health sector, many not yet so evident during Luhmann's lifetime, make it easy to grasp where their concerns originate: many doctors' consultations are for prophylaxis; health providers invest in prevention campaigns; exercise, diet, sleep routines and habit-forming behaviors are evaluated by whether they are “healthy”. The popularity of over-the-counter vitamins and holistic movements promoting “salutogenesis” as a more encompassing form of self-care than the orthodox conception of curation further illustrate this tendency.

When the correct demarcation of the system of medicine (or health) is thus debated within the theory, the debate mainly addresses the potential underrepresentation of “health”. While several authors feel troubled by Luhmann's relegation of health to a hollow “reflexion value” in a system exclusively focused on illness, the relation between science and medicine has hardly received any attention in these internal debates (Vogd, 2005 being a notable exception).

In this paper, I will focus on what a systems-theoretical approach can contribute to the sociological analysis of current developments in biomedical research—in particular, the alternatives it offers to the prevailing assumption of “blurring boundaries”. My aim is not to prove systems theory “right” by showing that medicine and science *are* closed systems. Neither, however, is the closed-but-coupled systems approach merely an alternative description of the same phenomenon from a different perspective. Analyzing the changes occurring in the contact zone between biomedical science and clinical medicine as responses to follow-up problems resulting from earlier evolutionary developments in this relationship, systems theory provides a clearer explanation for why these changes in biomedicine are occurring and why they are occurring *now*. The research on translational medicine and similar endeavors conducted in medical sociology and Science and Technology Studies provides rich empirical material. Interpreted through the lens of a theory of societal subsystems these studies can be situated within a broader account of the historical evolution of science and medicine.

Alongside several conceptual distinctions, I draw on one particular methodological instrument from the systems-theoretical toolbox: *systems comparison*. This approach “does not depend on a ‘similarity' of systems or even of their individual actions. […] The interest in a systems comparison rather results precisely in its ability to identify dissimilar aspects as equivalents” (Luhmann, 1974; p. 25). Following this methodological proposition, my aim is to “determine which aspects of reverence are problematic in the systems one compares”.

However, since the focus is on the relationship between science and medicine, here, the unit of comparison is not an isolated system. Instead, I compare intersystemic relations. One such relationship between two functional systems has received extensive attention in the writings of Luhmann and his followers: that between the political and the legal systems. The paper builds on Luhmann by using existing assessments of the politics-law relationship as a template for examining the analogous case of science and medicine. My first task, therefore, is to justify the selection of units of comparison (section 2). Then, some resulting structural features of the two systems-relationships are highlighted (section 3). Subsequently, following the program of systems comparison outlined by Luhmann, these structural features are observed as the products of the respective historical process of systems differentiation which starts from equivalent problem solutions (section 4). These analyses then provide the background for revisiting the supposedly blurred boundaries in contemporary high-profile biomedicine (section 5). The ample space given to analytically preparing this return to current biomedicine in the last section is also a deliberate contrast to the “blurring boundaries” literature whose method focusses on watching the actors very closely in their everyday practice. Here I argue that other things become visible when one first widens the perspective.

## 2 The units of comparison

The selection of the politics-law relationship as the basic unit of comparison is not arbitrary. It is justified by two structural features setting both compared constellations apart within the framework of functional differentiation and one methodological consideration.

Firstly, as will be argued more extensively in the following section, law and medicine are the two functional systems^4^ most exclusively based on conditional programs—they follow a strict if-then structure. This requires external references to select and justify the conditioning, which are found, respectively, in political legislation and scientific research results. No other functional systems rely to the same extent on conditionally programmed decisions that must be legitimated by external reference to other systems. This is further reinforced by the fact that, unlike other functional systems, medicine and the law do not operate through a fully independent symbolically generalized medium of communication (as the economy does through money or religion does through faith, for example). Instead, they rely more heavily on “secondary coding” of other media: political power in the case of law and scientific truth in the case of medicine. This accounts for a closer bond between politics-law and science-medicine than, for example, between politics and economy, where concerns are mostly directed at improper mutual interference, or between science and education, whose commingling mainly concerns their institutional unification under the roofs of universities.

As a consequence, these constellations generate dense coupling points that are easily interpreted as boundary erosion. Generally, the systemic borders identified by the theory largely coincide with those identified within the fields themselves. Indeed, systems theory is often accused of too readily assuming the existence of “the economy” or “the law” simply because people use the respective collective singulars (Kieserling, 2005). This is different for the systems with which we are dealing here. The separation between politics-law and medicine-(biomedical) science is more contested outside systems theory than the separation between other functional systems (with the potential exception of mass media and art). “Systems theorists”, Luhmann (1993, p. 407) notes, “generally assume that a distinction must be made between the legal system and the political system. They argue that these are different subsystems of the social system. This holds even more strongly if one accepts the concept of autopoiesis and insists on the autonomy and historical individuality of all social systems. However, this view is contested by others, likely the majority, due to the close and obvious connections between politics and law”^5^. A similar ambiguity besets the separation between science and medicine. In both cases, the contestation of systemic separations is not limited to theoretical debates (be it in political theory, social sciences, philosophy of medicine, science and technology studies, or other academic disciplines), but it also appears among the practitioners' self-descriptions. For politics and the law, Luhmann insists that “any systems theory paying attention to the meaning and mode of observation (distinction) of operations must accept that the political system and the legal system operate separately, that they are distinct systems—*even when the systems' self-descriptions contradict this*” (Luhmann, 2000, 390; his emphasis). We need not turn to the recent “blurring boundaries” debate to find the same discrepancy between self-descriptions and meaning processed through systemic operations in the case of medicine. While many physicians may consider their practice a craft, most will insist that medicine is (also) a science (Berg, 1995).

Secondly, note Luhmann's methodological considerations about systems comparisons: “Any systems comparison requires […] a preliminary theoretical analysis of the systems involved, clarifying their reference problems and choices of solutions. The comparison may yield different variations of a solution for one and the same problem of reference […]. The question of why the individual systems chose different variations then leads to concrete historical analysis” (Luhmann, 1974, p. 25). To meet these requirements, and given that we are comparing intersystemic relationships here, in section 4, I will argue that medicine and law evolved from an originally close proximity regarding their reference problems and that it was a historical, contingent-but-not-arbitrary development which led the law to externalize the justifications for its evolutionarily formed problem-solutions to politics, and medicine to externalize its justifications to science. There is a specific proximity between medicine and law resulting from their original functional equivalence as solution to the problem of explaining and resolving social deviance—a commonality that has been covered over time by successive layers of further structural evolution.

A third criterion for the selection of the given units of comparison concerns theoretical coherence: No other intersystemic relationship has been analyzed as extensively and thoroughly in systems theory as the one between the political and the legal system (especially Luhmann, 1993; p. 407–439; but also Stichweh, 1990; King and Thornhill, 2006; Luhmann, 1987, 1990c; Thornhill, 2018; Birle et al., 2012; Luhmann, 2015; Bora, 2003, to name just a few). Using the ample basis of concepts, theorems, and examples these provide ensures adherence to systems theoretical premises instead of introducing ad hoc concepts on which analogies are based.

## 3 Inter-systemic relationships

To outline several structural communalities between the two cases at hand, in a first step I draw on four concepts from Luhmann's vocabulary for describing functional systems—center/periphery-differentiation, contingency formulae, structural coupling, and conditional programs. The aim is not to check off items on a list of functional-systems characteristics but to show that the politics-law relationship as analyzed by Luhmann and the empirical reality of the modern science-medicine relationship share far-reaching analogies that make the former a useful lens for analyzing the latter.

The first theoretical concept to consider is the *center/periphery distinction*. Drawing on Shils (1961), Luhmann transfers this model—originally applied in world society theory—to the analysis of institutional arrangements within individual functional systems^6^. There it serves as a more adequate alternative to the hierarchy model of institutions. Perhaps not coincidentally, the two systems for which Luhmann elaborates this distinction are the legal (Luhmann, 1993, p. 321–337; Luhmann, 1990a) and the political system (Luhmann, 2000, p. 244–253), and he explicitly encourages the comparison with other functional systems^7^.

The theoretical problem addressed by this concept arises because some, but not all communications making up a functional system occur in organizations, and certain organizations have historically come to represent the system as a whole. In the case of law, these are courts; in the political system, state organizations. In their capacity as organizations in the systemic center, they are subject to an imperative of decision-making; they cannot not decide. The medical analogue of the legal courts—before which only a minority of legal disputes are brought, but which must process all that are—is the clinic, where only a minority of all cases of illness end up, yet all patients must be treated. Growing numbers of out-of-court settlements and outpatient-care solutions do not contradict but rather confirm the prohibitions against denial of justice and treatment: they function as compensatory mechanisms to manage case overloads without violating the imperative. By contrast, “private” practices are exempt from this imperative and thus belong to the periphery. Just as private contracts are consigned to the law's periphery and pressure groups to the political periphery, alternative medicine and household remedies are practiced in medicine's—making up the great majority of the respective systems' communications. Only the organized center molds legal issues and illnesses, respectively, into the form of “cases”, as which they receive their own recorded histories and formalized treatment procedures, documented in case files.

It is important to note that “centrality” in this framework must not be confused with “purity”. The most representative types of organization must not necessarily be the ones most exclusively taking decisions based on one specific code. Rather, all organizations display “multireferentiality” (Bora, 2001) and in some instances, organizations are even established with the concrete purpose of mediating between conflicting functional programs. In the present case of biomedical research, this applies especially to regulatory bodies such as the European Medicines Agency and the FDA in the United States. These organizations mediate between scientific validation and medical responsibility through codified standards without breaking down systemic differentiation.

Given the preceding discussion about the alleged overemphasis of illness at the expense of “health”, one might perhaps be tempted to identify the distinction between an organized center and a more eclectic periphery with that between medical and health-oriented action. This conclusion, however, would be premature. To see why, consider a second Luhmannian concept: that of *contingency formulae*. Luhmann uses this term to refer to specific normative semantics associated with functional systems (God for religion; scarcity for the economy; the common good for politics; limitationality for science etc.) to which they provide a basis of undeniability. In this role, they cut off demands for further justification without resorting to dogma. Contingency formulae function as core values that functional systems ascribe to themselves to maintain coherence among operations that could, in principle, be carried out differently.

There is good reason to assume that medicine's contingency formulae is “health” (Fuchs, 2006, p. 32ff.). The system can easily commit all its activities to this value. Its fundamental importance is almost impossible to deny (Schoene, 1963, p. 113ff.). Any critique of the system's structure must therefore take the form of criticism of the violation of the ideal of health-promotion (e.g., Illich, 1975)^8^, not against the ideal itself. For law, Luhmann introduces *justice* as the contingency formula answering to the question, “[h]ow can the system express its own *unity* in a normative program which, at the same time, can be applied *in* the system—*anywhere* in the system?” (Luhmann, 1993, p. 218). “Anywhere”, to link this to the previous discussion, includes both in the center and in the periphery. In this function of securing systemic unity despite internal differentiation^9^, contingency formulae and the center/periphery distinction complement each other (Luhmann, 2000, p. 249).

However, although all legal communication must be frameable as committed to the ideal of justice, not every attempt to achieve justice constitutes legal communication. Ascribing the label “illegal” to an (in)action can be perceived both as just or unjust, depending on the case; the systemic code requires a meaningful reflexion-value. The same holds for medicine and health. As “illegal” is not to be confused with “unjust”, neither is “ill” to be confused with “unhealthy”^10^, and the contingency formula applies for the whole system. Whichever stance one adopts in the debate over a “medical” vs. a “health system”, the center/periphery distinction cannot be used to argue for an organized center of medicine within a non-organized system of health (Bauch, 1996, p. 75–98; Hafen, 2009; Chávez and Mujica, 2022).

Turning back to what the center/periphery distinction reveals about the relationship between science and medicine, we find another piece of evidence for the autonomy of both systems in the organization of their centers. As noted, the center/hierarchy differentiation constitutes a second-order form of differentiation in that it allows systemic unity despite (internal secondary) differentiation. The law's internal segmentation is closely linked to the political segmentation into national states. While impressing structural constraints on the legal system, the formation of nation states in the seventeenth and eighteenth centuries also provided an evolutionary boost to the self-organization of legal reasoning (Stichweh, 1990), so that the structural isomorphy allowed operational autonomy. By contrast, the internal segmentation of medicine is more independent from the segmentation of science into disciplines. The institutionalization of the clinic in the eighteenth century not only produced distinctions between hospital physicians and ambulatory practitioners (Mayntz et al., 1988, p. 128), but also tied this distinction to a process of professional specialization guided by a complex interplay between nosological categories and histological demarcations (Pinell and Brossat, 1988, p. 580; Rosenberg and Golden, 1992; Stichweh, 1994, p. 312). This histological structuration, in turn, was tightly followed by technological developments (Atkinson, 1995; 61f., 88). There are general hospitals and there are clinics specializing in particular diseases or organs, and within them nurses, surgeons, radiologists, and others enact their own division of labor. Medicine's internal differentiation is heterogenous, combining multiple principles of structuration, and remaining clearly independent of any inner-scientific differentiation. In the resulting arrangement, all medical subsystems are particularly geared toward legitimation from one specific scientific discipline, namely biology—as the political domain relevant to the law is legislation.

The emergence of such independent structures of internal differentiation presupposes that the system can afford a considerable degree of indifference toward its environment, an improbable condition taken for itself, since functional specialization simultaneously increases reliance on the resources of other systems. Gene therapy for sickle cell anemia could not be risked without economic investments or legal provisions for the case of failure. As is well known, Luhmann borrowed the concept of *structural coupling* from Humberto Maturana to describe how systems can react to the activity of other systems without compromising their own operational closure. When particular types of irritation from the environment keep recurring, mechanisms tend to emerge to ensure that mutual reference can occur beyond the singularity of specific situations. The politics-law relationship is again the best documented example, with constitutions serving as the primary mechanism for structural coupling (Luhmann, 1990c). The mechanisms of structural coupling between medicine and science are, I argue, more recent and less firmly institutionalized than constitutions. Yet, they fulfill a comparable function in producing information which can be treated both as an instruction for medical practice and as a scientific truth, generalized beyond particular situations and cases. This function is performed primarily through *trials* and *models*.

Clinical trials were introduced in the interwar period with the declared aim of integrating science with medical care, replacing to a degree the system of legitimation via the professional authority of the physician (Armstrong, 1977). Trials are statistical experiments assessing the effectiveness of drugs, diagnostic procedures, or technological devices before they are permitted into the clinic. They follow methodological prescriptions including, among other things, double-blind designs and comparison with a control group (receiving a placebo or standard of care). While inscription into a clinical trial often serves as a last resort action for medicine which, as noted, must find ways of acting even when “nothing more can be done”, for biomedical research, the same practice implies that patients are turned into a (scarce) resource (cf. Keating and Cambrosio, 2007, p. 215f.).

Models, on the other hand, are abstractions in the medium of causality that represent isolated connections between causes and effects. One strand of the literature on models asks what purpose they fulfill and typically arrives at two types of answers. It highlights either their role as representations (abstractions in the factual dimension) or their axiomatic function as procedural heuristics (abstractions in the temporal dimension) (Godfrey-Smith, 2006; Wernecke, 1994; Giere, 1999)^11^. A second strand examines how models work and often emphasizes their intermediate position between theory and experiment or data (Morrison and Morgan, 1999; Keller, 2000; Rosenblueth and Wiener, 1945). Since this literature originates predominantly in philosophy of science, these accounts focus on the use of models on the scientific side of the structural coupling. The representational aspect involves hetero-reference, that is, access to the structure of “reality” (not just “pure” data), as the model stands for something outside science. Their characteristic in-between status, on the other hand, connecting theory and methods, allows translating the reality into the self-referential programming of the scientific code (cf. Luhmann, 1990b, p. 392–432 on theory and methods as conditional programs).

A further distinction frequently invoked in discussions of models is that between explanation and prediction (Morton, 1993; Shmueli, 2010). Here the perspective shifts from an exclusive focus on the scientific side of modelling to its role as an enabler of structural coupling with other systems. From a theoretical point of view, prediction need not be understood as the forecasting of concrete outcomes. More abstractly, it can refer to the possibility of manipulating the time dimension which is inaccessible in the natural system that the model represents. To understand what happens in a natural system, one must conduct experiments and wait and see. The formalized system of the models offers an alternative: it enables inferences which eliminate the time component—that is, predictions (cf. Rosen, 1985). In biomedical research, the most important models are *animal models* (Ankeny, 2007; Lewis et al., 2012; Strasser, 2019, p. 29–66). In their case, too, the predictive aspect of their function must not be underestimated. These models are not mere stand-in for humans, used because these are either too complex or out of reach for ethical reasons. They also allow answering what-if questions by looking ahead.

Temporality thus plays a central role in structural coupling. Luhmann accordingly describes it as a form of coordinating systems that develop their individual *eigen*-temporalities. In the present case of coupling between science and medicine this aspect is particularly important, given the medical system's extraordinary propensity to urgency. Illness is the state in which a change in status takes precedence over everything else. As a social system, medicine's function can be described to a degree as re-synchronizing the absolute acuteness of the situation with the social environment.

Trials use statistics in the medium of quantification, while models use mechanisms in the medium of causality, both creating the “laboratory conditions” that designate their outcomes as scientific. To find gene therapies for sickle cell anemia, for instance, numerous clinical trials have been conducted and thousands of mice transplanted with edited genes (Ballantine and Tisdale, 2025). Contemporary medical research, as published in journals and presented at conferences, consists almost exclusively of combinations or serializations of models and trials.

In this process, scientific programs can be coordinated with medical ones. Trial designs must fulfill “scientific” standards aimed at ensuring accuracy, reliability, validity, and reproducibility. They must also ensure patient safety and a standard of care that does not undercut baseline treatment, thus coordinating scientific accountability and methodological standards with clinical responsibility and patient welfare. This transition is broken down into partial steps such as trial phases or the distinction between in silico, *in vitro*, and *in vivo* models—each subject to specific regulations with different degrees of rigor.

To reconnect this discussion to the center/periphery differentiation it is useful to have a second look at the analogous case of constitutions in politics and law. The legal system, Luhmann writes “changes the law in light of future court decisions and then conforms to the prevailing law, which in turn can give rise to opportunities for observation and reasons to amend the law. To differentiate the conditionalities of this decision-making process (and only for that purpose!), this system describes itself as a hierarchy—whether of institutions or of norms”. At the turn to the nineteenth century, in science, under the guise of “theory,” a hierarchy of knowledge forms and epistemological sources is being replaced by a system of scientific propositions within individual disciplines (which now exist side by side), while, at the same time, in law, under the guise of “dogmatics,” a corresponding replacement is taking place: the hierarchy of legal sources is being supplanted by a hierarchy of legal rules within a heterarchical system of sources (Stichweh, 1990, p. 265; Teubner, 1996). Institutionally, the legal center is much more hierarchically organized than the medical, where individual clinics and practitioners tend to pride themselves on their autonomy. Nevertheless, in cases where decisions cannot follow the routine of what has always been done in these cases—in the context of rare cases or the application of newly introduced diagnostic or therapeutic devices—lately, in the context of the vastly influential “evidence-based medicine” movement aiming to “integrating individual clinical expertise with the best available external clinical evidence from systematic research” (Sackett et al., 1996, p. 71), an “evidence hierarchy” has been established in medical self-description with large-scale statistical trials on top (Clarke et al., 2014; Worrall, 2007)^12^. Especially where the change of decision premises is at stake, the system describes itself as a hierarchy—just as Luhmann observes for the law. These are self-descriptions, however. While we may be “deceived by our fascination with the constitution and its proclaimed values, the image of a hierarchically guaranteed supreme authority” (Luhmann, 1990c, p. 215), the center/periphery distinction was introduced precisely to account for the replacement of hierarchical structures in modernity. What places constitutional courts and governments, as well as biomedical research centers and university clinics at the representative center of their functional systems is not the dependence of all communications and decisions on deductions from highest principles. Rather, it is their prerogative to preside over the change and changeability of the premises of decision-making.

At this point, another similarity between law-politics and medicine-science comes into view, setting both these intersystemic relationships apart from those between other functional systems. Structural couplings exist between many systems. What binds politics and law on the one hand and medicine and science on the other closer together than other systems is an arrangement of mutual externalization of legitimation. To present this point, a fourth and final concept must be introduced: Luhmann distinguishes between goal-oriented and conditional programs (Luhmann, 1964). The former are based on means-ends reasoning, the latter on if-then considerations. It may be natural, at first glance, to view medical treatment as a goal-oriented endeavor. After all, curation and the attenuation of suffering are the unquestioned aim of all medical activity. From a theoretical perspective (see also Vogd, 2005, p. 240f.), however, this is a misconception. If medicine were to directly orient its decision-making in the form of goal-directed means-ends programs, it would have to resort to comprehensive clinical outcome measurement. This would be technically impossible, due to the unmanageable dispersion of causality. In the clinical practice, it is possible only in an absolute minority of cases to control whether and to what degree a given health outcome, positive or negative, was due to a medical intervention. It would also be disastrous in terms of medical legitimation: the illusion of the effectiveness of medical interventions would be shattered. Again, we find a parallel to the law, where the operational functioning of the system is based on the shared assumption that the system is operationally functional, not just independent of enforcement rates, but despite them (Popitz, 1968; Hofmann, 2022). The solution consists in holding clinical trials *prior to* the approval of the drug or technology, and under controlled experimental conditions. These serve not only to comply with methodological scientific standards of reliability, objectivity, and reproducibility, but also to allow their results to be translated into *conditional programs* for medical decision-making. The law is the paradigmatic case of a system based on conditional programs (Luhmann, 1993, p. 195–204) and it adapts these programs based on stimuli from political legislation. Medical conditional programs that are legitimized by scientific research and channeled through standardized procedures, are the direct complement. Physicians defend diagnoses and therapies based on scientific evidence; and where the quest for knowledge is insufficient to justify scientific research, the prospect of saving lives lends it additional importance. Symptoms, signs, and test results inform a diagnosis or verdict, which conditions a treatment selection. In both cases—laws conditioning legal decision programs as well as scientific evidence conditioning medical decision programs—general rules must be fitted to individual cases, which are inherently more complex (denser in detail) than what provisions can regulate. Judicial review procedures stabilize the conditioning in legislation and jurisdiction while ethics committees, regulatory agencies, and trial governance bodies assume the analogous role in biomedicine.

This is not to say that the medical periphery forgoes the benefits of scientific explanations and justifications. Luhmann even conceives of the periphery as a “contact zone with other functional systems” (Luhmann, 1993, p. 322). The healing powers of household remedies are more often explained by the biochemical effects of vitamins than by magic and homeopathic reasoning rests on elaborate systems that, while perhaps challenging “school medicine”, claim access to truth. What distinguishes these types of scientific orientation is the degree of formalization, generating a greater need for scientific/political legitimation in the legal/medical center, where decisions must withstand potential appeals.

Given this arrangement, the relationship between science and medicine is undoubtedly more asymmetrical than the analogous case of mutual externalization between politics and the law. Nevertheless, the notion of medicine as an applied science should not be used to reduce the mutual legitimization arrangement to unilaterality. In the following sections thus, I will argue, respectively, that medical-pathological considerations decisively influenced the development of physiology and biology and that several of the phenomena described as “blurring boundaries” can be described as the results of a new prominence of reciprocity within this coupling.

## 4 Evolution and differentiation

As systems comparison is rooted in the analysis of common reference problem, I now outline, first, to which problem medicine and the law originally offered alternative responses and how they differentiated in the course of social evolution, and second, how medicine's specific coupling with science—historically more contingent than our familiarity with the arrangement commonly allows us to see—developed.

### 4.1 Medicine and law: patterns of per-differentiation

For many centuries, medicine maintained close ties not only with science, but also with religion. That it should also be closely associated with the law may initially be surprising. The association becomes clearer, however, when one looks at criminality and sickness. As the sociology of deviance and the sociology of roles have long argued (Parsons, 1951, p. 312–315; Schoene, 1958, p. 85–88; Aubert, 1982; Lorber, 1967, p. 303ff.), both constitute a breach of social expectations. Consequently, in segmentary societies, where illness is considered to be the effect of retaliatory magic, the ways of “treating” both tend to be extremely similar. In this context, “diagnosis” amounts to identifying the “responsible” person or entity (Laín Entralgo, 1958; Evans-Pritchard, 1937). Anthropological evidence suggests that expert roles—judges and healers—are often only rudimentarily differentiated in these social contexts (Postert, 2004; Platenkamp, 2015, p. 91ff.). Following a general pattern (Luhmann, 1975b, p. 153f.), we can assume that their differentiation is preceded by differentiation of situations occuring when the moral responsibility for changing the deviant behavior is attributed to different points of reference, depending on the types of case. To this day, when we encounter the violation of a social norm, we may not always be sure whether to criminalize or to pathologize the behavior^13^. The difference between the two lies in the type of response. Deviance is criminality whenever “normative expectations” are upheld and the responsibility for change is put on the offender; it is illness where the behavior cannot be ascribed to “ill will”, but must be attributed to the deviant's bodily, mental or social environment (Simon, 1993). Even where such differentiation has occurred, medical and legal attempts at remedying the situation use similar techniques. In ancient Greek, *pharmakon* can take both the meaning of remedy and penalty. Medicine and the law thus share an initial concern with protecting the community from the destructive potential of deviance.

In both cases, the differentiation of expert roles appears to presuppose an increasing separation between diagnosis and treatment—an important prerequisite for the establishment of conditional programs and differentiation of externalization addresses. As long as the judgment *is* the treatment, it can be enforced by the affected parties or the community (without a political monopoly of power or a diagnostic procedure based on physiological theories). By contrast, both physicians and judges divide their tasks into finding the source of a malady and subsequently choosing an adequate cure or penalty. Among the oldest extant written sources not only legal codes are particularly prominent, but also medical sources. Both are based on conditionality (if-then), reflected in the textual form (protasis-apodosis). This, in turn, enables the emergence of casuistry (see, for example, Buchheim, 1965; Geller, 2010, 2004; Maul, 2007; Böck, 2014). The forensic procedure, culminating in the declaration of the expert judgment which often includes the selection of a penalty or treatment, tends to be standardized into processes that manifestly serve to ensure the correctness of the judgment and latently function to secure acceptance among the affected parties (Luhmann, 2017)—the roots of *legitimization*. The casuistic processing via deductive reasoning we tend to associate with doctor-patient interaction can be interpreted as a type of interrogation, for which standard procedures result as a product of learning in the medieval universities. Their higher faculties of medicine and law become the remit of corresponding professions in early modernity. Here, the specific form of boundary-work (Gieryn, 1983) of lawyers and physicians takes off.

It is, however, a significant step from institutions for the processing of cases, to the idea that ensuring justice and health in the community should be done ex officio. While a “prosecuting society” (Moore, 2007) aimed at the prevention of both, criminality and disease (describing both in terms of “purity” and “contagion”) already exists in medieval cities, the idea that criminal prosecution should be the responsibility of political authorities emerges in early modernity, at the same time as health politics, both under the label *policey*. This is particularly significant for the present interests, because it creates the preconditions for the formation of organizations that mediate between functional spheres. In the eighteenth century, the prison is turned from a place where criminals await the trial into that where penitence turns them back into ordinary citizens; while the hospital is turned from a place where the dying await their end to that where therapy turns them back into ordinary citizens. The prison and the clinic are born (Foucault, 1975, 1983) to manage and temporalize the exclusion of the criminal and the sick, as the class-dependency of health and legal provisions is reduced and functional differentiation starts to force society into promoting “universal inclusion” into its partial systems (Stichweh, 2024). As in the new organizations, cases can be not only processed but more easily counted and compared, the ground is prepared for a statistically based trial system. The fields from which the first attempts to formalize probability had drawn in the seventeenth century were law and medicine, as areas in which probability is gradable (Schneider, 1981, p. 12ff.). As political arithmetic is applied to medicine and the law, the secondary disciplines of epidemiology and criminology begin to complement their traditional sphere of knowledge-generation. Both focus on populations and prevention, thus compensating for the inevitable focus on individual cases and reactive problem-solving genuine to the two corresponding professions. In both cases, the application of statistics promotes searching the environment for more distant causes of criminality and sickness behind the effective causes or motives identified in diagnostic and legal proceedings. This extension of the classical sphere of medical and legal influence in contexts of medicalization and securitization recently seems to bring both fields into close proximity again, as technological progress has bred visions of biosocial control and risk management in which the potentially-ill and the potentially-criminal are subject to “biosecurity” measures that are not always easily distinguishable from each other (Rose, 2001; Braun, 2007). The medical domain most involved in these developments is molecular medicine (“molecularization of life”), the legal one is transnationality (“extraterritoriality”)—I will come back to both developments as cases of particularly strained structural coupling.

### 4.2 Medicine and science: dependence through interdependence

Perhaps the most central prerequisite for the liaison between science and medicine as closed-but-coupled systems, is standardized disease classification. Until the Renaissance, diagnosis mainly involved identifying the problem. Diagnosis as the correct naming of the disease, on the other hand, becomes an autonomous aspect of medicine during the 18^th^ century, as nosology emerges (Coste, 2015). The transition to standardized diagnostic practice can be dated to the beginning of the 19^th^ century. This was driven by the engagement with epidemic diseases (cholera, yellow fever, smallpox), which were easily recognizable as uniform pathologies across large regions, and it was epistemologically supported (not caused, as is often assumed) by the success of pathogen theory in the second half of the 19^th^ century (Rosenberg, 2002a). The reification of disease creates generalized entities around which medicine can organize its self-reference and once illness has become a positively identifiable entity, it can start to act as the positive code value. As medicine increasingly centers around the ill/health code, it detaches from the distinction living/non-living. The predecessor of medical research, physiology—conceived as a general *science de l'homme* responsible for physical, pathological, and ethical questions—drifts away from medicine, finding a home instead in the emerging scientific discipline of biology. Medicine can now be separated from but coupled with this discipline.

However, despite far-reaching commonalities between medicine and the law, the above analogies also have limitations. One such limitation lies in the fact that they overrepresent a specific area within the law as the point of reference for comparison: the description of the legal evolution, starting from the problem of deviance, focused predominantly on *criminal* law. While it is common—not only in popular thought, but occasionally also in the sociology of law with its root in theories of social control—to treat as “the law” what empirically constitutes only a small part of legal interactions, from a theoretical point of view this is problematic. Luhmann himself repeatedly warns against mistaking the highly representative subfield for the entirety of the legal system. Moreover, his sociology of law draws a significant part of its uniqueness from how serious he takes the insight that the law works best when it remains virtual and invisible. A lack of enforcement, if it is due to a lack of perceived need thereof, is the surest sign of law's social force—a force built on the substitution of conditioned expectations-of-expectations for concrete actions, that frees up resources and space for evolutionary variation.

What does this imply for the comparison with medicine? On the level of theory construction, one possible conclusion would be to return to the speculations about a “medical subsystem” *within* a “functional system of health”. If the analogy was selective regarding the legal system, maybe the same applies for the health system: medicine might be understood as one particularly representative but not exclusive, subfield—as criminal law is to the legal system. In this view, medical treatment would be the analogue of law enforcement, that is, the organizationally structured part of the system that steps in once the first line of defense, made up of expectations, fails. The *function* of a health system could accordingly be modelled after that of the law, described by systems theory as the counterfactual insurance of normative expectations. Medicine provides the security that, in case someone gets ill, something can and will be done to remedy the situation.

An alternative way of accounting for this ambivalence theoretically would be by questioning the strictly binary logic presupposed by Luhmann's description. Thus, drawing on fuzzy-logic, Kron and Winter (2005) have proposed to introduce two types of vagueness: “vagueness of coding” where it is unclear which code value must be applied and “vagueness of belonging” where it is unclear whether a communication is part of the system or not. For the case of illness and health, the first of these vaguenesses has been defended (and formalized in fuzzy-logic) by Sadegh-Zadeh (2000). The latter vagueness could be used to describe the system's periphery.

Rather than arguing directly for or against these theoretical options^14^, I propose an alternative theoretical interpretation of the parallels between medicine and the law. On the one hand, the symmetry of their initial settings—the equivalence of explanations for deviance and of ascriptions of responsibility for addressing it—does not imply that the functional systems emerging from it should also develop in perfect analogy. Their respective societal environments, including their relationship to other functional systems with distinct evolutionary trajectories and functions, preclude such symmetry. On the other hand, said initial setting was not completely symmetric to begin with. To expand on these points, it is useful to return from the comparison between law and medicine to that between the law-politics and the medicine-science relationship.

Science is the social field most prominently tasked with producing explanations. More specifically, it explains the normal state of the world—initially in contrast to theological metaphysics concerned with the supernatural, and later by formulating general principles and laws of nature. The deviations ascribed either to social disruptions via the law or to physical and psychological disruptions via medicine are deviations from the normality science explains. While the law opposes medicine on the axis separating normative from cognitive expectations, science opposes it on the axis separating the normal from the pathological. The relationship between the law and politics is different in this regard. The law is “positive but potential” (the standard value is “lawful”, interventions are usually only threatened), while medicine is “negative but actual” (the standard value is illness, intervention is acute). One consequence consists in a medical preference for false positive errors as opposed to the legal preference for false negative errors (Rizzi and Pedersen, 1992; Stratton, 2015). If, against this background, we extend the analogy between legal and medical differentiation to that of their respective relations with politics and science, we encounter in both cases a particularly strong pattern of “increasing differentiation through increasing interdependence”. The “rule of law” emerges out of Enlightenment political philosophy and formalizes the law in a process that substitutes positive, changeable law for the combination of the natural law doctrine and legal pluralism. Professionalized medicine, analogously, emerges from the “scientific revolution” in a process that substitutes biomedicine for the combination of natural philosophy and medical pluralism. In both cases the above-described arrangement—externalization of legitimation for conditional programs—emerges. While the law bases its validity on political power, medical decision-making grounds its reliability on scientific truth. The fact that the medium of power is oriented on acting, while the medium of truth is oriented on experiencing (Luhmann, 1975a) can account for the difference in scope between the medical system and the law—which is more than merely criminal law.

Something else can be drawn from this interpretation if we focus especially on “positivization”. Structural couplings are structural in that they can be used repeatedly across a range of different situations. To fulfill this role, they must be able to combine stability with flexibility. Both the politico-legal coupling via constitutions and the bio-medical coupling via models and trials are “positive”, in that they are instituted through social procedures, rather than by divine or natural necessity; they can be changed without losing legitimacy, provided established procedures are respected. Consider, again, sickle cell anemia. As in the case of cancer, the pathology gets its name from the visual resemblance between one of its manifestations and an otherwise unrelated object. Yet, while cancer received its name in antiquity from surface appearances, the “medical gaze” had to penetrate the patient body and pass through the microscope before the sickle-like shape of certain blood cells could become salient in early twentieth century. This is not the only difference between the shape metaphors: While modern physicians would not assume that the cancer-like outline of the tumor explains anything about the pathological process underlying it, the sickle-shape is understood to share at least a cause with the mechanism of pathogenesis. Nevertheless, in both cases the name is that of a class of variations of a disease. In the current version of the *International Classification of Disease* issued by the WHO, sickle cell anemia appears under chapter 3A51, with different sigla specifying various subspecies. Diagnosis takes the form of a classification (Jutel, 2011).

This is a modern phenomenon. While diagnosis, in the sense of etiological inquiry (into responsible evil spirits), is nearly as old as medicine itself, well into the early modern period, diagnostic expertise consisted primarily in the detailed outline of the physiology behind the pathogenic process. Only in the eighteenth century—not only under the influence of the period's notorious enthusiasm for taxonomy, but on a more fundamental level as a long-term consequences of the printing press—did diagnosis assume the meaning of giving a name to the disease—not an individual name, but that of a disease class (Stolberg, 2021, p. 155–224; Rosenberg, 2002a, 2007; Coste, 2015). The decisive aspect here, regarding the development of structural coupling and conditional programs, is the possibility of separating more strictly than before between diagnosis and treatment-selection—a separation enabling variability of subsequent recombination. There are cases in which diagnostic classification is shaped by available treatments (Rasmussen et al., 2024; Pot, 2025), yet it remains a striking feature of modern medicine how autonomous diagnosis and treatment—originally the names of standard textbooks sections—are. Both externalize their justifications to science, but they do so independently. This way, the conditioning between them, the selection of a drug or procedure given a certain diagnosis that has been selected from a pool of existing disease categories given a number of signs, symptoms and test results, remains the exclusive domain of medicine while coupling with science is maintained. The relative independence between diagnosis and therapy is illustrated by the century that elapsed between the naming of sickle cell anemia and the approval of gene therapies for it. Nosology-based classificatory diagnosis, I suggest, is the medical equivalent of the positivization of law. This leads us back to the discussion of blurring boundaries.

## 5 Discussion: blurring boundaries?

I have described the relationship between medicine and science in systems-theoretical terms and traced it as the product of a long process of evolution and differentiation. Bringing these aspects together allows us to reinterpret the current “blurring boundaries” debate as a moment within that historical process.

Historicizing this discussion, Warner (1995) argues that by drawing the conventional distinction between science and medicine along the lines of the laboratory and the clinic, amounts to buying into a nineteenth-century science-policy construct: according to him, the notion that clinical medicine cannot be scientific, but at best applies scientific findings, emerged from a campaign aimed at securing laboratory medicine its own independent place among the experimental sciences. Warner proposes therefore to take seriously the fact that clinicians throughout history have perceived their work as scientific and, to account for this, to move away from speaking of “science” in the (collective) singular (see also Sturdy, 2011). In the light of the above considerations on structural coupling, systems theory, which not only deliberately speaks of “science” in the singular but also ascribes systemic status to it, suggests the opposite reaction to the same assessment of a problematic separation: Not everything that happens in laboratories is science. Much of it belongs to medicine insofar as it fulfills the function of generating clinically applicable conditional programs.

The boundaries that are supposedly becoming muddled between biomedical science and clinical care can be broadly classified into three categories, depending on which type of entities they separate (not anymore): roles, organizations, and “social worlds”. The emblematic figure representing the first category is the “clinician-scientist”, professionals trained—often in specially designed university and medical school programs offering combined degrees—to translate research findings into clinical practice. Originally conceived as the evidence-based medicine movement started questioning the decision-making capacities of physicians based merely on professional experience and authority. After falling out of the spotlight during the 1990s, this figure has recently experienced a strong renaissance in the context of translational medicine (Lander and Atkinson-Grosjean, 2011; Vignola-Gagné, 2014). Here, the sociology of professions and especially its concept of “hinges between ecologies” (Abbott, 2016) provides a formulation of the coupling between domains perfectly compatible with systems-theoretical notions of structural coupling. Two aspects should be noted however regarding this figure: firstly, the exact translational tasks to be fulfilled by clinician-scientists still often underdetermined (Hendriks et al., 2019). In practice, their work often consists in tasks of both clinicians and scientists, as well as their coordination, rather than introducing genuinely new tasks. This composed role thus, secondly, often even helps maintain the role separation—as the title suggests—even though both roles are bundled in one person.

The second type of entity in which boundaries are perceived to become permeable are certain organizations specifically designed to enable multiple functional references. In research hospitals patients are also study participants, to shorten turnaround times, many care centers nowadays count with in-house laboratories, and with Translational Medicine a broad spectrum of new institutional entities, such as molecular tumor boards—interdisciplinary teams in cancer centers elaborating treatment recommendations based on genomic test results—has been introduced to bridge the gap between laboratory and clinic. Here, the structural coupling approach complements the more empirical descriptions found in the science and technology studies literature. Theoretically, organizations are the most central locus for structural coupling, as they mold functional exigencies into decision programs (Lieckweg, 2001). Empirically, this is presented in the need to balance trial inclusion criteria with best-interest care, study endpoints with ethical norms of compassionate continuation and the like. Sometimes the regulatory standards of one system can be used to synchronously achieve goals in the other, for instance when study enrollment gives patients access to off-label therapies. The fact that these practices require the existence of different sets of rules and programs lends evidence to the assumption that both spheres remain separate.

“Social worlds” theory, lastly, is the theoretical background against which the concept of “boundary objects” was originally formulated (Star and Griesemer, 1989). Although this concept is more malleable (more entities can be considered boundary objects than can serve as coupling mechanisms), a particularly fruitful theoretical synergy can come from this concept's emphasis on the bilateral referenciability. Indee, what many of the “blurring boundaries” approaches generally argue for is more recognition of the mutual influence between medical and scientific considerations—against the notion of a unilateral transfer from scientific insights to clinical application. While an asymmetry in the legitimation relationship cannot be denied, a long tradition going back (at least) to Canguilhem's (1966) analysis of the relationship between pathology and physiology—recently taken up, for example, by Keating and Cambrosio (2003)—argues that the importance of medical developments and considerations in guiding research programs and procedures often goes unnoticed. Which questions are asked, which molecular pathways are analyzed, which models are used in research—all this is conditioned on the aim to find cures (Lewis et al., 2014).

What still requires further explanation is the *current* proliferation of blurring boundaries observations. This phenomenon, too, has a parallel in the politics-law relationship. Both a “politization of law” and a “judicialization of politics” (Domingo, 2004) are attested especially in analyses of recent developments in international and “transnational law”, that is, in an area where structural coupling between both systems is precarious (Teubner, 1997). Some systems-theorists argue that principles such as obligations *erga omnes* and *ius cogens* are functionally equivalent to constitutions beyond the national-state (Fischer-Lescano, 2005; Brunkhorst, 2007). Regardless of how one evaluates such claims, it can safely be stated that “world law” is notoriously prone to suspicions of “politization”. A similar dynamic can be observed in recent developments of biomedicine, above all the sweeping computization, datafication and algorithmization of biology spreading to medical research, have compromised the stability of the model and trial system (Le Tourneau et al., 2014; Superchi et al., 2022; Keating and Cambrosio, 2012). Not coincidentally, the earliest demands during the institutionalization of translational medicine were directed at reform and innovation of the coupling mechanisms between biomedical research and medical care: they claimed more flexibility in trial design and more studies on human, in contrast to animal, disease biology (Marincola, 2003; Zerhouni, 2009). Mouse models cannot represent the complexity of human molecular pathways adequately; the increasing personalization of targeted drugs makes classical patient stratification little feasible and presupposes dynamic adaption of study criteria; *in silico* trials simulate outcomes, but the indispensable use of algorithms and big data requires bigger study populations than traditional trials. Classificatory diagnosis, identified above as a key driver of the differentiation between medicine and science, loses importance to predictions based on molecular alterations (Armstrong, 2019). Also, the temporal coordination afforded by structural coupling comes under stress in precision medicine, which is especially timing-sensitive while at the same time relying on molecular test results whose turnaround time cannot be compressed at will. All these developments are follow-up problems resulting from a mismatch between newer developments and the mechanisms originally established to regulate the channels between science and medicine.

The reduced stability of the structural coupling mechanisms is one feature shared between developments in transnational law and in cutting-edge, especially precision-, medicine. Two further features deserve attention, in the light of the previous analysis. One concerns the differentiation between diagnosis and therapy in medicine and between codification and enforcement in law. In both spaces of supposedly weakened systemic boundaries, this distinction is subject to considerable pressure from both centrifugal and centripetal forces.

In molecular medicine, the gap between growing numbers of risk-factors and available biomarkers on the one hand and effective, approved drugs on the other is widening. At the same time, classical disease categories lose importance when machine learning algorithms infer disease risks or origins from associations between molecular data points and suggest therapies not only based on the disease classification but also on predicted therapy effects (Armstrong, 2019; Petersen and Ursin, 2025). Meanwhile, in transnational law, the *lex mercatoria* enacts law without codification; and human rights courts issue judgments without compliance (Berger, 1999; Teubner, 2000; Neves, 2005; Kastner, 2017). In both domains, one response to the unstable relation with political power and scientific knowledge has been an increased recourse to prevention (Opitz, 2011; Chatterjee et al., 2016; Nagel et al., 2017).

Finally, both areas of supposedly blurring boundaries are among those where the most innovation takes place and where standards and routines are the least established. In many respects, the organizations, in which biomedical and legal novelties are produced, are accordingly understood as testing grounds—not least in their own self-descriptions. As contact zones between functional systems, these organizations are especially sensitive to irritations. At the structural level, new types of organizations are experimented with (arbitration boards, Truth and Reconciliation Commissions, Molecular Tumor Boards) and integrated into extended chains of decision-making. The considerable effort invested in developing new routines becomes visible in new institutional formats. Hetero-observations, not least important among them sociological ethnographies “following the actors”, are particularly prone to finding systems logics mixed in interactions and organizations' day-to-day operations. This should not, however, be confused with the dissolution of the differentiation between operational logics of functional systems.

## 6 Conclusion

Systems(-relations) comparison provides a structural explanation for the empirically observable phenomenon underlying the blurring boundaries debate. It emphasizes the stress that biomedical innovation puts on existing forms of structural coupling, which is experienced within the system through mounting difficulties of conditionally programming treatment-selections based on diagnostic classifications and to which the system reacts, currently, especially by creating new types of mediating organizations tasked with the formalization of new practices.

Modern biomedicine is characterized by the bilateral contact between patients in need of acute—and often expensive—care and a machinery for producing scientific novelty, both operating under strict ethical and regulatory oversight. This bilateral arrangement shares structural features with other organizations situated in the contact zones between functional spheres. Central banks, for example, mediate between political and economic programs, and research developing technologies with military applications face considerable scrutiny. Yet coupling in biomedical research is unusually dense. While the comparison with the law-politics relationship as the “most similar case” provided the conceptual means to account for some of this intensity, what it did not cover highlights the uniqueness of the case: unlike organizations such as universities coupling education and science without one existing for the sake of the other, the interface between medicine and biology is structured around mutual externalization. Structural coupling, while necessary in all functional systems' relations to their environments, is here particularly *structural*, in the sense that its institutionalization exceeds punctual links and switching between functional programs takes a processual form: in the precision medicine “pipeline”, scientific criteria tend to dominate “upstream” (Nelson et al., 2014; Cambrosio et al., 2024), while clinical programs gain prominence “downstream”.

In this paper, a highly abstract theoretical framework was confronted with concrete and institutionally distinctive empirical developments. While the theory operates with the very broad category of the functional system of science, the empirical case draws its force from biomedicine. The observations developed here should not be uncritically generalized to all of science. At the same time, the asymmetry between the structural analysis at the level of societal subsystems (“science”, “medicine”, “the law”) and local practices within specialized subdisciplines also speaks to a theoretical claim. While most social studies of biomedicine investigate empirical reality through thick descriptions, Luhmannian theory of functional differentiation, rather than merely describing the clinician's everyday conflicts between patient care and scientific curiosity in a different vocabulary, foregrounds the social and historical conditions underlying them. Systems comparison is a methodological means to this end. From this point of view, strained coupling is unlikely to result in ever more boundary blurring, or even dissolution between medical decision-making and biological research. Given their evolutionarily stabilized ‘dependence-through-independence' the emergence of new coupling mechanisms is more plausible; some of which are already becoming visible.

## Data Availability

The original contributions presented in the study are included in the article/supplementary material, further inquiries can be directed to the corresponding author.

## Footnotes

1 This is not to say that pathophysiology downstream from the genetic etiology is simple. It involves multiple processes and clinical manifestations vary.

2
https://www.nytimes.com/2024/10/21/health/sickle-cell-disease-gene-therapy-patient.html

3 Throughout this paper, I use the term “systems theory” to refer to Luhmann's version and the elaborations following in his wake.

4 In systems theory, the “canonical” functional systems are the economy, science, law, art, mass media, politics, religion, education, medicine, sports, and intimate relations – several others are debated.

5 All translations from German are mine.

6 On the level of the theory of society, Luhmann's assumption is that the description of world society in terms of center and periphery is outdated, given the primacy of functional differentiation. However, not just despite, but precisely because of this primacy (which is not a substitution), the center/periphery differentiation can plausibly be assumed to continue as a secondary differentiation within the individual functional systems.

7 See also Fuchs (2001) for science, which he conceives, however, as a network rather than a system.

8 Illich's critique of “medical pathogenesis” is essentially the articulation of the fundamental paradox of medicine: how to know whether distinguishing between ill and healthy is itself healthy or pathological? Following Luhmann, the function of the contingency formula is exactly to hide this foundational paradox.

9 Put in other words: It is the principle of differentiation between differentiated (center) and undifferentiated (periphery).

10 A linguistic ambiguity of the term “healthy” is responsible for some part of the confusion here. Most Western languages have conflated what in Latin is distinguished as *sanus* and *salubilis*. Foucault (1977, p. 103) makes a similar observation in a talk that was translated from Spanish (where *sano* is different from *saludable*) into French for *Dits et écrits III* (*Salubrité* and *santé*, p. 222f., being less faithful to the everyday meanings of *salubridad* and *salud* than phonetics suggests). The same confusion does not occur in the law, because *just* and *legal* are obviously distinct words. Nevertheless, the German “zu(un)recht”, applying to all kinds of justifications, not just legal ones, has also led to some confusion (e.g. Clam, 2006, p. 85f).

11 There are also models focused on the social dimension of meaning, we could call them “role models”. Daston (2022, p. 23–47) describes their background as one of the original meanings of the concept of rules. They seem to be less significant for science, however, than e.g. for ethics, sports, and mass media.

12 See Eisenberg (2001) on the differences between what medicine and the law treat as evidence.

13 On the history of the patient's moral responsibility for her illness see Temkin (1949), Rosenberg (2007), and Kühn (2001).

14 In any event, such interpretations would have to answer some theoretical questions, among them how to account for the code inversion within the medical system, as theorized by Luhmann (2005).
